# World Health Organization Recommends Comprehensive School Health Services and Provides a Menu of Interventions

**DOI:** 10.1016/j.jadohealth.2021.04.036

**Published:** 2021-08

**Authors:** David A. Ross, Mary Louisa Plummer, Paul Montgomery, Kid Kohl, Nandi Siegfried, Elizabeth Saewyc, Valentina Baltag

**Affiliations:** a Department of Maternal, Newborn, Child and Adolescent Health and Ageing, World Health Organization, Geneva, Switzerland; b Department of Social Policy, Sociology and Criminology, School of Social Policy, Muirhead Tower, University of Birmingham, Edgbaston, Birmingham, UK; c University of British Columbia School of Nursing, Vancouver, British Columbia, Canada

Schools have unmatched potential to provide health services to older children and adolescents. Nowadays, in virtually every country of the world, the great majority of school-age children and adolescents (5–19 years) attend school on approximately half of the days of the year. Global net primary and secondary school enrolment rates^1^ have increased substantially over recent decades; by 2020, they were estimated to have reached 89% and 66%, respectively [1,2]. In countries in the Organisation for Economic Co-operation and Development, children and adolescents are estimated to spend an average of 7,590 hours in the classroom over the 8–10 years that they are in primary and lower secondary school [3]. School health services may be the only institutional way to meet the health-care needs of most school-age children and adolescents on a regular basis and at scale [4]. School health services also have the potential to increase health equity through improved access to services because they are usually free at the point of use and are provided within, or very close to, the school. This can be especially important for underserved children and adolescents who do not have adequate coverage with effective health services for their needs. Furthermore, when implemented with reasonable quality, school health services are highly valued by students, parents, and communities [5].

Despite all these potential advantages and the fact that most countries have established school health service programs, these programs generally have not received the attention they deserve from researchers, policy-makers, and development partners [6]. In high-income countries, school health services often rely on a network of school nurses, sometimes including school-based health centers [4,7]. However, in many low- or middle-income countries, school health service programs are more severely underfunded and/or delivered with limited reach and scope [7]. In practice, in numerous low- or middle-income countries, school health services are limited to those that can be delivered by teachers, such as counseling or periodic deworming, and/or to rare visits by clinical staff from a local health facility, for example, to administer human papillomavirus vaccinations. This situation represents a critical missed opportunity. It means that adolescents in these contexts will often only contact health services if they are ill or injured, and with delay, when they are severely ill. Furthermore, adolescence is a key period for the onset of many health concerns, such as mental health or visual acuity disorders. It is also when different kinds of risk behaviors that have major impacts on future adult mortality and morbidity are either initiated or consolidated, such as the use of alcohol, tobacco and other substances, risky sexual behaviors, and the adoption of healthy or unhealthy dietary and exercise habits [8].

In 1995, the World Health Organization (WHO) launched the Global School Health Initiative, which later developed into the multiagency Focusing Resources on Effective School Health (FRESH) Initiative. This defined six interrelated pillars of a health-promoting school [9]:

1. Healthy school policies
2. Physical school environment
3. Social school environment
4. Health skills and education
5. Links with parents and community
6. Access to (school) health services

Until now, there have not been any global recommendations on what the health services pillar should include. In an important new development, WHO has published its first-ever guideline on school health services [10]. The guideline is based on systematic reviews of the literature on the effectiveness, feasibility, and acceptability of comprehensive or multicomponent school health services. It also draws on a review of global WHO publications to identify health service interventions for 5- to-19-year-olds for consideration, followed by a global survey of 442 school health experts on the relative suitability of these interventions for inclusion in school health services. This evidence base informed the guideline as it was developed by school health experts within WHO and UNESCO, with the support of a diverse guideline development group of 16 academics, national policy-makers, and program managers representing all regions of the world, with inputs from six independent reviewers.

The guideline makes a strong recommendation that comprehensive school health services should be implemented in schools.

It goes on to provide a menu of 87 specific interventions categorized as essential or suitable for inclusion within school health services either everywhere (e.g., provision of first aid, promotion of menstrual hygiene management) or in certain geographical contexts only (e.g., micronutrient supplementation, promotion of insecticide-treated bednets). The menu is organized as a matrix by eight health areas:

1. General/cross-cutting
2. Positive health and development
3. Unintentional injury
4. Violence
5. Sexual and reproductive health (including HIV)
6. Communicable diseases
7. Noncommunicable diseases, sensory functions, physical disability, oral health, nutrition, and physical activity
8. Mental health, substance use, and self-harm and by seven types of health activity:
1. Health promotion
2. Health education
3. Screening leading to care and/or referral and support, as appropriate
4. Preventive interventions
5. Clinical assessment leading to care and/or referral and support, as appropriate
6. Health services management
7. Support for other pillars of a health-promoting school

The guideline includes a compendium of excerpts from WHO source documents related to each of the 87 interventions so that readers can see the basis for their inclusion.

The aim is that this guideline will be the first in a series of detailed global guidance documents on school health service programing and implementation that will be produced by WHO in the coming years. It is part of the resource package to inform the new WHO/UNESCO initiative *Make Every School a Health Promoting School* through a standard-driven approach [11].

This guideline comes at a unique time in history, when the COVID-19 pandemic has highlighted the vital link between educational institutions and health. One of many effects of school closures or shifts to remote learning during the pandemic has been studentś reduced access to school health services. This is likely to have had a particularly harsh impact on vulnerable and/or underserved children and adolescents, which makes it even more critical that adequately resourced and well-implemented school health services are part of efforts to “build back better” after the pandemic.

The WHO guideline on school health services represents the results of over two yearś work by WHO and UNESCO staff, academics, policy-makers, and program implementers. The evidence reviewed during the guideline's development shows that if comprehensive school health services are implemented well, they will be well-accepted and will bring important benefits for students. The guideline provides unambiguous support for comprehensive school health services. It will promote the implementation of evidence-based services through its menu of interventions, will strengthen the school nursing and school health professions around the world, and ultimately should contribute to improvements in the health and well-being of children and adolescents globally.

## Footnotes

1 The net school enrolment rate is the number of students of official school age who are enrolled in education as a percentage of the total children of the official school-age population.
